# miR-130-3p Promotes MTX-Induced Immune Killing of Hepatocellular Carcinoma Cells by Targeting EPHB4

**DOI:** 10.1155/2021/4650794

**Published:** 2021-07-23

**Authors:** Liangtian Shao, Qing Ye, Moyang Jia

**Affiliations:** ^1^The Second Affiliated Hospital of Shandong First Medical University, Tai'an 271000, Shandong, China; ^2^Taian Maternal and Child Health Hospital, Tai'an 271000, Shandong, China

## Abstract

The vast majority of primary hepatocellular cancer is hepatocellular carcinomas (HCCs). Currently, HCC is one of the more common cancers in humans, and it has a high mortality and disability rate. Mitoxantrone (MTX) is an antitumor drug that can block type II topoisomerase. It has been reported that immunogenic cell death evoked by MTX can induce the discharge of damage associated with molecular patterns (DAMPs) and subsequently influence immune cell infiltration in the tumor microenvironment. High mobilities aggregation box 1 (HMGB1) is the prototypical extracellular DAMP. Many cellular processes have been reported to involve EPHB4 receptor tyrosine kinases, but the relation of DAMP and EPHB4 is uncertain. In this research, we assessed the impact of miR-130-3p by Edu incorporation test on cell proliferation, and we have proven its impact on HCC cell migration through Transwell and wound healing tests. Flow cytometry was applied to study its influence on apoptosis. Luciferase report test was integrated in detecting the miR-130-3p target gene. The influence of miR-130-3p on the manifestation of classical DAMPs was studied, such as HMGB1, ATP, and Calreticulin. A coculture experiment was carried out to further confirm its effects on immune cell infiltration. The result displayed that miR-130-3p overexpression considerably facilitates apoptosis and suppresses the migration or proliferation of HCC cells. EPHB4 was confirmed as the target gene of miR-130-3p. Overexpression of this target gene promotes emission of Calreticulin, ATP, and HMGB1 and subsequently promotes DCs maturation and proliferation of CD4+ T cells. In summary, our results demonstrated that miR-130-3p inhibits HCC cell proliferation and migration by targeting EPHB4 and promotes drug-induced immunogenic cell death.

## 1. Introduction

Primary liver cancer is predominantly hepatocellular carcinoma [1–3], which is currently the leading cause of cancer worldwide. Traditional therapeutic strategies for HCC include surgery, radiotherapy, and chemotherapy [4, 5]. However, the effect of this treatment is not satisfactory.

Mitoxantrone, also known as MTX, is an Adriamycin analogue that was developed in the 1980s to specifically inhibit type II topoisomerase based on its antitumor properties [6]. It is often employed for the management of various cancer types, including acute leukemia, breast cancer, myeloma, and lymphomas [7].

Most importantly, MTX can induce immunogenic cell death and promote immune cell infiltration. Immunogenic cell death is known to induce adaptive immunity against dead cell antigens, especially when they are released from cancer cells [8–10].

DAMPs are signals that can be released or exposed when dying, stressed, or injured cells are present. These molecules often play the role of danger signals or adjuvants, thus helping the immune system [11]. HMGB1 is a highly conserved nuclear protein that senses and coordinates the stress response of cells. It plays an especially important part not only as it was intracellularly a defender of DNA chaperones, apoptosis, and autophagy, but also outside the cell as the prototypic DAMP. Classical DAMPs include ATP and HMGB1, and the DAMPs advance the development of dendritic cells and further activate the immune system [12].

EPHB4, a part of the biggest group of receptor tyrosine kinases-protein tyrosine kinases is ordinarily communicated on neurons and endothelial cells, where it plays a vital part in the vascular era and organizes gathering.

EPHB4 is a constituent of the biggest family of receptor tyrosine kinases. It is often manifested as a protein tyrosine kinase on endothelial and neuronal cells and has a significant role in multiple cellular processes including cell growth, survival, and angiogenesis. Its receptor could be a tyrosine kinase, which is included in numerous cellular forms such as angiogenesis, cell development, and survival.

A few reports propose that EPHB4 is overexpressed in different cancers, counting breast, prostate, and colon [13, 14]. MicroRNAs are evolutionarily conserved small RNAs that do not code and which are useful in almost all bioprocesses by targeting mRNAs [15, 16]. A new study suggests that miRNA dysregulation is included in tumor movement [17]. Nevertheless, the potential part played by miR-130-3p in HCC cells still remains unknown. MiR-130 was first found in regulating respiratory syndrome virus replication and porcine reproduction. A previous study demonstrated that the downregulation of miR-130 contributed to the activation of lung fibroblasts by targeting IGF-1. A previous study demonstrated that miR-130 was served as a tumor promotive miRNA in most human cancer.

In this study, our research was aimed at exploring the embedded mechanism on molecules of miR-130-3p-EPHB4 in terms of transfer of MTX-induced immunogenicity in hepatocellular carcinoma cells, as well as providing any potential effective targets of therapy for PC treatment.

## 2. Materials and Methods

### 2.1. Patients and Tumor Samples

Our experimental samples were all provided by the Department of the Second Affiliated Hospital of Shandong First Medical University. We obtained approval letters from the University's ethics committee. Our conducted study was also aligned with Helsinki Declaration in terms of the guidelines and principles. We examined the samples (patients) by PCR and explored the pattern of expression in EPHB4 in liver cancer tissues and regular controls with the help of TANRIC software platform.

### 2.2. Cell Lines and Antibodies for Immunoblotting

The HepG2 cells employed in the experiment were acquired from ATCC Cell Bank (Manassas, VA, USA). Under suitable conditions of 5% CO_2_ and 37°C, these cell cultures are cultured in Dulbecco's modified Eagle medium (DMEM). The first antibody (CST, Trask Lane Danvers, MA) was a rabbit polyclonal antibody at a dilution of 1 : 1000. The first antibodies are Cleaved Caspase-3, AIFM1, Pro-Caspase-3, and Bcl-2. The secondary antibody is the anti-rabbit polyclonal antibody GADPH (CST, Trask Lane Danvers, MA) at a dilution of 1 : 2000.

### 2.3. In Vitro Cell Multiplication Measure

96-well plates with a density of 5,000 cells/well in the total medium were used to inoculate the cells. After 24 hours of cell culture, the cells became precipitated with 4% formaldehyde and recolored with 0.5% crystal violet. Finally, the crystal violet was dissolved in 10% acetic acid and cells were tested for transfection at *λ* = 580 nm using an ELx800 spectrophotometer after 24 and 48 h, respectively.

### 2.4. Plasmid Development and Transfection

We transfected inhibitors and mimics of miR-130-3p using Lipofectamine 3000, which was sourced from Life Technologies. The concentration of the solution was set at 20 nM according to the instructions from the manufacturer. Based on the expression of green fluorescent protein (GFP), we harvested and cultured cells after sorting lentivirus-infected cells with MoFlo XDP (Beckman, USA) for subsequent functional studies. In addition, to explore whether miR-130-3p could regulate EPHB4 manifestation by directly binding to the established 3′ UTR sequence, we established luciferase reporter plasmids that had binding sites or wild-type for EPHB4. To induce the overexpression of circ-EPHB4, we cloned the coding sequence into the pEX-3 vector (Shanghai Gene Pharma Co., Ltd.), respectively. We transfected the above vectors with Lipofectamine® 3000 (Invitrogen, Thermo Fisher Scientific, USA) based on the instructions of the manufacturer. Following a 48 hour transfection, the efficiency of each group of cells was identified using qRT-PCR.

### 2.5. Public Clinical Datasets

We obtained immune infiltration information associated with EPHB4 expression from the TIMER2.0 online dataset.

### 2.6. Wound Healing Test

After the cells have grown all over the cell vial, a fine needle is scraped in one direction over the cell monolayer. Wounded cell monolayers were washed with phosphate-buffered saline to remove cellular debris. We analyzed the wound healing and migration of cells in the remaining slits at different time points by using an inverted microscope.

### 2.7. Western Blotting Analysis

Based on the instructions of the manufacturer, we extracted the total protein from each group. The selected protein assay kit was then employed in determining the concentration of total cellular proteins. Samples of denatured protein were denatured at 100°C for 10 min and then analyzed on 10% SDS-PAGE gels and moved to PVDF membranes (Millipore, MA, USA) through electroblotting. The membrane was closed with 5% skim milk for 1 h at room temperature before incubating them overnight using primary antibody at 4°C. Then, a secondary antibody was used in incubating the membrane after its washing (Proteintech) for 2 hours at room temperature. Finally, the immunoblots were visualized and analyzed with an ECL chemiluminescence detection system (Thermo Scientific), and the relative integrated density values (IDVs) were determined using GAPDH as an internal control. The assessments were performed in three samples. The main antibodies included the following: IKK*β* (1 : 1000; Cell Signaling Technology, USA); GAPDH (1 : 6000, Sigma, St. Louis, MO, USA).

### 2.8. ATP Detection

Intracellular and extracellular ATP concentrations were measured by utilizing the fluorescein-based ENLITEN ATP test (Promega, Madison, WI, USA).

### 2.9. Stream Cytometry Examination

The processing of cells was achieved through trypsin before being washed using PBS. Apoptosis tests were performed utilizing Annexin V-FITC Apoptosis Detection Kit (Beyotime). According to the instructions provided by the manufacturer, anaplastic cells were dual-stained with PI and Annexin V-FITC utilizing the Annexin V/FITC kit (Thermo Logical, Shanghai, China). Analysis was performed on a BDTM LSRII stream cytometer (BD Biosciences).

### 2.10. Luciferase Columnist Test

Firefly correspondent quality expression vectors driven by SV40 enhancers were acquired from Gene Copeia. 3′-UTR arrangements of wild-type or mutant EPHB4 were included downstream of the luciferase quality, where no oligonucleotides were included within the control vector. Renilla luciferase was utilized as a marker to screen transfection productivity. The luciferase movement was measured with a discuss photo-switch reagent.

### 2.11. Statistical Analysis

All collected data are expressed as mean ± SEM. A single evaluation of variance via ANOVA used to be carried out using SPSS 21.0 (SPSS Inc., Chicago, Illinois, USA) for assessment between groups. We also performed pairwise comparisons between groups using the Student-Newman-Coors (SNK) test. The statistical results with *P* values of below 0.05 were identified as significant, with significant differences.

## 3. Results

### 3.1. miR-130-3p Suppresses Restraint of Movement and Overgrowth of HCC Cells

**Figure 1:**
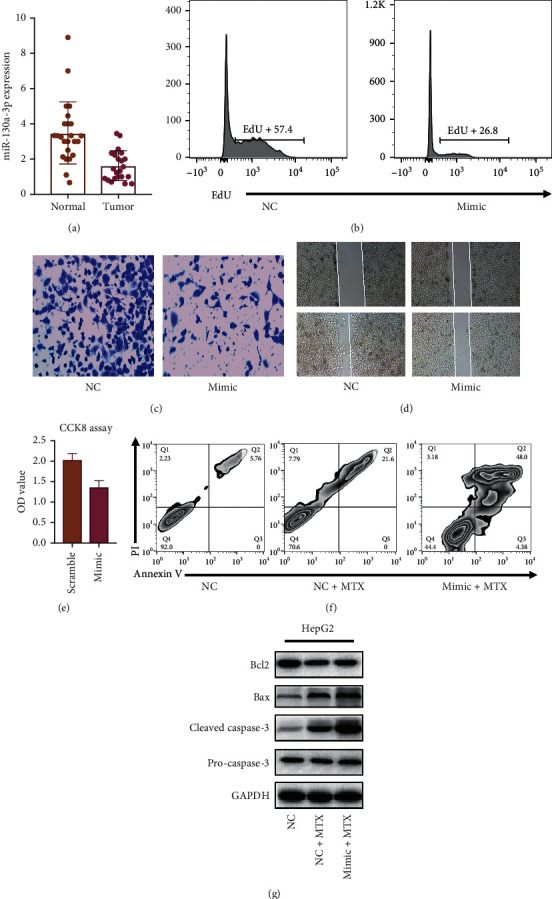
miR-130-3p suppresses migration and proliferation of HCC cells. (a) CR evaluation of miR-130a-3p expression in HCC tumor tissues and regular tissues adjoining to cancer. (b) EdU binding assay after transfection of HCC cells with NC and mimic. (c) Transwell assay after transfection of HCC cells with NC and mimic. (d) Wound healing test of HCC cells after transfection of HCC cells with NC and mimic. (e) CCK8 assay after therapy of HCC cells with MTX for 24 hours. (f) AnnexinV analysis of apoptosis rate. (g) Western blot evaluation of molecules linked to the apoptotic pathway.

The miR-130-3p expression in the clinical sample was confirmed by PCR. As shown in Figure 1(a), HCC tumor tissue expresses lower miR-130-3p than paracancer normal tissue, implying that miR-130-3p may serve as a tumor-suppression effect. To affirm the function of miR-130-3p of HCC cells, EdU incorporation assay was confirmed. As shown in Figure 1(b), after the transfection of miR-130-3p mimic, the percentage of EdU positive cells decreased, showing that miR-130-3p suppressed HCC proliferation. To explore the impacts of miR-130-3p on HCC cell migration, a Transwell assay was performed. As shown in Figure 1(c), after the transfection of miR-130-3p mimic, migrated cancer cells decreased significantly. The result showed that miR-130-3p suppresses the migration of HCC cells. To further confirm this conclusion, a wound healing assay was performed. As shown in Figure 1(d), the result confirmed our previous conclusion that miR-130-3p overexpression impaired HCC cells migration. We further confirmed the impacts of miR-130-3p on the overgrowth of cells. As shown in Figure 1(e), the CCK8 assay indicated that after transfection of mimic, the proliferation of tumor cells was suppressed. To confirm if miR-130-3p overexpression promotes MTX-induced apoptosis, flow cytometry was used. As shown in Figure 1(f), 12 treatments of MTX increased the apoptosis rate of HCC cells. After miR-130-3p overexpression, the apoptosis rate was further enhanced. Western blot was conducted to investigate the underlying mechanism. As shown in Figure 1(g), Bcl-2 was suppressed by MTX, Bax, and cleaved caspase-3 expression was enhanced by MTX treatment. After the transfection of miR-130-3p mimic, the expression of cleaved caspase3 was further enhanced. Those results indicated that the overexpression of miR-130-3p promotes MTX-induced apoptosis.

Our result affirmed that miR-130-3p suppresses the migration and proliferation of HCC cells, and the overexpression of miR-130-3p promotes MTX-induced apoptosis.

### 3.2. miR-130-3p Targeting EPHB4 and Promotes Apoptosis of HCC Cells

**Figure 2:**
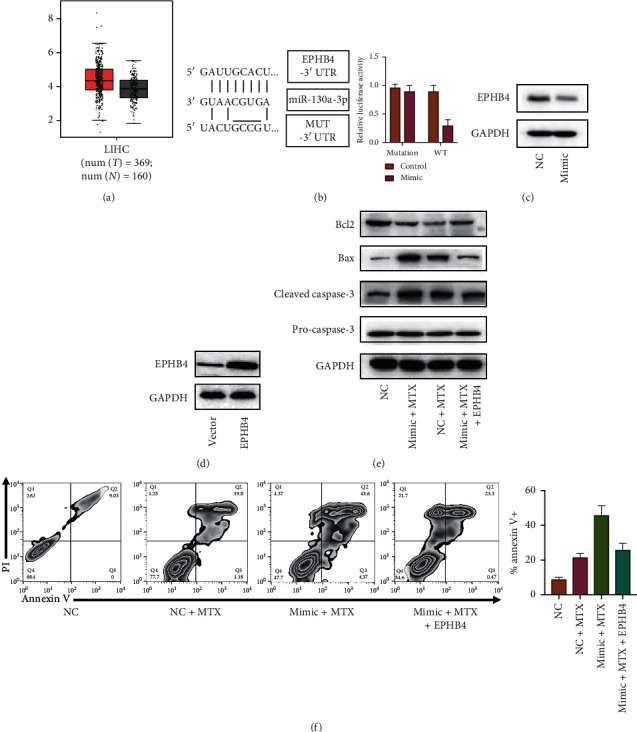
miR-130-3p targets EPHB4 and promotes apoptosis of HCC cells. (a) EPHB4 expression in HCC cancer tissue and normal tissue. (b) Luciferase assay was used to evaluated the relationship between miR-130-3p and mRNA of EPHB4. (c) Correlation between miR-130a-3p expression and EPHB4 expression. (d) Immunoblot evaluation of EPHB4 expression after transfection with mock or NC. (e) Immunoblot evaluation of EPHB4 expression after overexpression vector. (f) Western blot analysis of apoptosis-associated molecular. (g) AnnexinV analysis of apoptosis rate.

To confirm target gene of miR-130-3p, TargetScan dataset was used. Given the dataset, EPHB4 may act as the target protein of miR-130-3p. As shown in Figure 2(a), the *d* showed that EPHB4 expresses higher in tumor tissue relative to normal tissue, indicating that EPHB4 may function as a tumor-promote gene. As shown in Figure 1(c), the online dataset showed that the expression of EPHB4 is negatively associated with the expression of miR-130a-3p in HCC. After the overexpression of miR-130-3p, as shown in Figure 2(d), EPHB4 expression was limited, indicating that miR-130-3p expression may regulate EPHB4 expression. To further confirm that EPHB4 is a direct target gene for miR-130-3p, a luciferase report measure was undertaken. Wild-type EPHB4 3′-UTR and mutant EPHB4 3′-UTR with nucleotide substitution within the putative binding site were built, and after that, subcloned into luciferase columnist vectors. As displayed in Figure 2(b), the transfection of miR-130-3p significantly suppresses luciferase activity, and insignificant differences were observed in luciferase with mutating UTR.

We are curious about whether the effects of miR-130-3p are through the regulation of EPHB4. To answer this question, the development of the EPHB4 overexpression vector and the effects of EPHB4 overexpression were evaluated by western blot. As shown in Figure 2(e), the result indicated that EPHB4 was successfully overexpressed. The mechanism of miR-130-3p-induced effects was further studied. As shown in Figure 2(f), as previously mentioned, the integration of MTX enhances the manifestation of Bax and cleaved caspase-3, and the transfection of miR-130-3p mimic further promotes these effects. But after transfection of the EPHB4 overexpression vector, miR-130-3p induced apoptosis was partly inhibited. The effect was also affirmed by flow cytometry, as depicted in Figure 2(f); overexpression of EPHB4 reversed miR-130-3p induced apoptosis. These results indicated that miR-130-3p promotes the apoptosis of HCC cells through the downregulation of EPHB4.

Taken together, our result indicated that miR-130-3p targets EPHB4 and promotes the apoptosis of HCC cells.

### 3.3. miR-130-3p Overexpression Promotes Emission of DAMPs upon MTX Treatment

**Figure 3:**
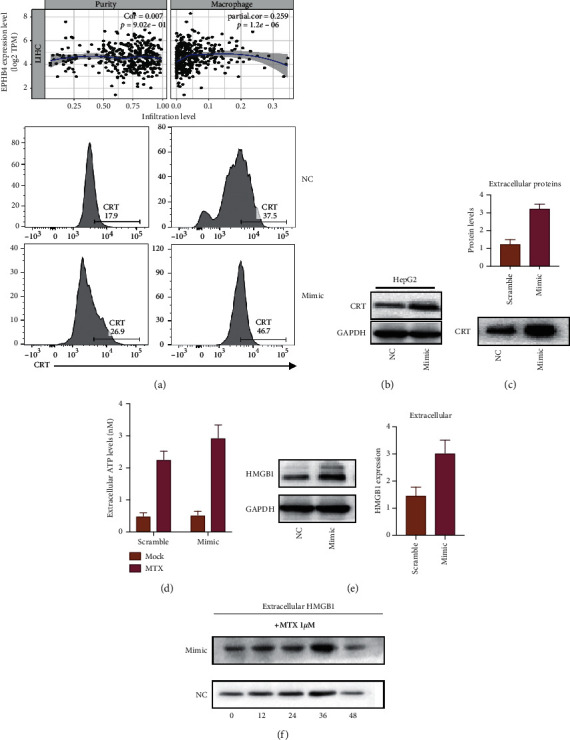
miR-130-3p overexpression promotes emission of DAMPs upon MTX treatment. The best segment shows the affiliation of safe cell invasion with EPHB4 expression. (b) Western smudge investigation of calreticulin after overexpression of miR-130-3p. (c) Immunodetection of CRT within the medium and extracellular calmodulin communicated by entirety cell lysis. (d) The energy of initiated ATP discharge. (e) Immunodetection of extracellular HMGB1 and HMGB1 in medium. (f) Time course of HMGB1 discharge after MTX treatment. Blunder bars imply ± s.d.

Based on the prediction of the TIMER dataset, EPHB4 expression is associated with the alternation of immune cell infiltration, including macrophages, as shown in Figure 3(a). We next study the effects of miR-130-3p on immune cells. Immunogenic cell death is a cell death that does stimulate an immune response by the emission of DAMPs. Classical DAMPs include ATP, high mobility group protein B, and calreticulin, and miR-130-3p may influence the emission of DAMPs and subsequently affect immune cells. As shown in Figure 3(a), the calreticulin expression in the cell surface was detected by flow cytometry. Our results showed that MTX treatment greatly increased the expression of calmodulin on the cell surface. After the overexpression of miR-130-3p, it was further increased. Interestingly, the basement expression of calreticulin was moreover expanded after the transfection of the miR-130-3p mirror. As shown in Figure 3(b), calreticulin expression in HCC cells was evaluated by western blot. After the transfection of mimic, the calreticulin expression in HCC cells was increased. We then tested the amount of calreticulin that was released by HCC cells after MTX treatment. As shown in Figure 3(c), secreted calreticulin that was induced by MTX treatment was increased as miR-130-3p was overexpressed in HCC cells.

As illustrated in Figure 3(d), the kinetics of ATP release in cell culture medium showed that cells overexpressing miR-130-3p had a stronger response to MTX's along with an increase in the amount of ATP secreted. HMGB1 expression was also evaluated. As shown in Figure 3(e), after miR-130-3p overexpression, the total expression of HMGB1 and its secretion were increased. As shown in Figure 3(f), the temporal profile of the HMGB1 release indicated that the HMGB1 release was slightly amplified by MTX treatment. miR-130-3p overexpression generally amplified extracellular HMGB1.

In conclusion, our results confirm that overexpression of miR-130-3p promotes the release of DAMPs when MTX is treated.

### 3.4. miR-130-3p Overexpression Promotes T-Cell Proliferation and DC Maturation

It has been reported that the DAMPs advance the further active immune system and the maturation of dendritic cells. In order to confirm whether miR-130-3p overexpression induced DAMPs influence activation of immune cells, HCC cells were transfected with miR-130-3p mimic. NC served as control and was treated with MTX for 12 h and cocultured with human youthful DCs for 24 hours.

**Figure 4:**
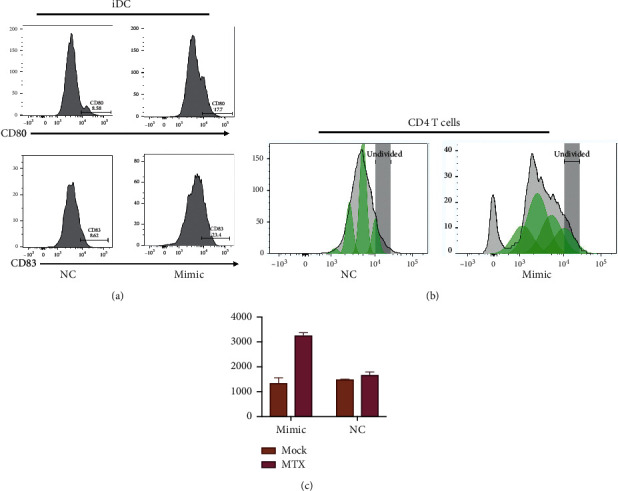
miR-130-3p overexpression promotes DC maturation and T-cell proliferation. (a) Investigation of expression of CD86 and CD80 by flow cytometry following DC coculture with cancer cells. (b) Analysis of CD4 T-cell proliferation by CFSE following treatment with CM acquired from tumour cells cultured with DC. (c) IFN expression in conditioned medium of CD4+ T cells by ELISA.

As shown in Figure 4(a), after DC was cocultured with HCC cells and miR-130-3p overexpression, the maturation marker of DCs such as CD80 and CD83 was increased compared with the coculture and HCC cells without miR-130-3p overexpression.

Phagocytosis of dying tumor cells and mature DCs play an active immune role, so we stimulated the proliferation of T-cell receptors (CD3/CD28) using a medium with DCs cocultured with tumor cells under such conditions. As shown in Figure 4(b), the conditioned medium derived from coculture of DCs and HCC cells with miR-130-3p overexpression significantly promotes the proliferation of CD4+ T cells. The released IFN from CD4+ T cells was evaluated by ELISA. As shown in Figure 4(c), the conditioned medium derived from the coculture of DCs and HCC cells with miR-130-3p overexpression promotes the IFN production from CD4+ T cells.

Our result indicated that miR-130-3p overexpression promotes DC maturation and T-cell proliferation.

## 4. Discussion

Liver cancer is one of the common cancers in humans. It has the characteristics of a high fatality rate and high disability rate. Despite the continuous development of surgical technology, it is still a medical problem [18]. Chemotherapy is the traditional remedy approach for hepatocellular carcinoma, and MTX is one of the most secure and most famous anticancer marketers at therapeutically utilized doses and has anticancer outcomes on a range of hepatocellular carcinoma cellphone lines, inclusive of HepG2, MHCC97, Huh7, and Morris 5123 cells.

For the patient with advanced HCC, MTX did now not exhibit a favorable tumor response or normal survival. However, the combination of MTX with other chemotherapy strategies is under investigation [19, 20].

EphB4 has been detailed in that EphB4 includes a potential oncogenic part and is a critical controller of essential physiological and pathophysiological forms, such as tissue designing, amid advancement, angiogenesis, and tumor movement. It has been reported that EPHB4 overexpression is related to destitute forecast in patients with ovarian cancer. The overexpression of EPHB4 in colorectal cancer cells promotes its proliferation and migration, invasion, and angiogenesis [21, 22]. EphB4 expression was too essentially higher in delicate tissue sarcomas, and mRNA and protein expression were altogether expanded in synovial sarcomas, while the regulation mechanism of EPHB4 still remains unknown [23]. In this experiment, the Edu incorporation assay method was conducted to detect the effects of miR-130-3p on cell proliferation. At the same time, we also detected the effect of HCC cell migration by the Transwell method and other experiments. Flow cytometry was performed to study its influence on apoptosis. Luciferase report assay was carried out to confirm the target gene of miR-130-3p. The influence of miR-130-3p on the expression of classical DAMPs such as Calreticulin, ATP, and HMGB1 was studied. A coculture experiment was used to further confirm its effects on immune cells infiltration.

In this study, we confirmed that EPHB4 is a target gene of miR-130-3p. After miR-130-3p was overexpressed, the migration and proliferation of HCC cells were significantly inhibited. miR-130-3p overexpression also promotes MTX-induced cancer cell apoptosis. After EPHB4 was reexpressed, miR-130-3p induced proapoptosis effect was partly inhibited, indicating that miR-130-3p promotes MTX-induced apoptosis through the downregulation of EPHB4.

It has been reported that MTX treatment can induce Immunogenic Cell Death (ICD) melanoma, osteosarcoma, and mouse colon cancer cells, acting through an eIF2*α* phosphorylation-dependent mechanism [24, 25]. Immunogenic cell death is the effect. Dying cancer cell triggers robust immunological memory and immune response [26]. It has been reported that ICD is actuated by harm related atomic designs (DAMPs) and the outflow of numerous DAMPs. Cell surface presentation to CALR, extracellular discharge of ATP, and the atomic protein HMGB1 are considered to be all-inclusive donors to nearly all ICD sorts. Subsequently, these DAMPs are commonly utilized as markers for in vitro evaluation of ICD inducers.

In this basic study of liver cancer, we confirmed that after miR-130-3p increased, and the expression of calmodulin on the cell surface also increased significantly. After the transfection of mimic, the total calreticulin expression in HCC cells was increased. We then tested the amount of calreticulin that is released by HCC cells after MTX treatment. Secreted calreticulin that was induced by MTX treatment was increased because miR-130-3p was overexpressed in HCC cells. After miR-130-3p overexpression, the total expression of HMGB1 and its secretion were all increased. The increased emission of DAMPs increased DCs maturation and subsequently promoted CD4+ T-cell proliferation. Increased IFN secretion was also observed when CD4+ T cells were treated by condition medium derived from the coculture of DCs and HCC cells with miR-130-3p overexpression.

In summary, our results confirm that miR-130-3p inhibits the proliferation and migration of HCC cells by targeting EPHB4 and induces immune death of hepatocellular carcinoma cells by promoting drug.

Nonetheless, there are several limitations to the present study. The study did not investigate the association between other MircroRNAs and primary hepatocellular cancer and any other potential interactions between genes. Therefore, future studies will focus on the interactions between microRNAs.

## Data Availability

The data of this article are included in pictures and illustrations. The datasets used and analyzed during the current study are available from the corresponding author upon reasonable request.

## References

[1] Chedid M. F., Kruel C. R. P., Pinto M. A. (2017). Hepatocellular carcinoma: diagnosis and operative management. *ABCD. Arquivos Brasileiros de Cirurgia Digestiva (São Paulo)*.

[2] Kanematsu M., Semelka R. C., Osada S., Amaoka N. (2005). Magnetic resonance imaging and expression of vascular endothelial growth factor in hepatocellular nodules in cirrhosis and hepatocellular carcinomas. *Topics in Magnetic Resonance Imaging : TMRI*.

[3] Sun H., Song T. (2015). Hepatocellular carcinoma: advances in diagnostic imaging. *Drug Discoveries & Therapeutics*.

[4] Shah S. S., Wu T.-T., Torbenson M. S., Chandan V. S. (2017). Aberrant CDX2 expression in hepatocellular carcinomas: an important diagnostic pitfall. *Human Pathology*.

[5] Rhee H., Nahm J. H., Kim H. (2016). Poor outcome of hepatocellular carcinoma with stemness marker under hypoxia: resistance to transarterial chemoembolization. *Modern Pathology*.

[6] Xie B., He X., Guo G. (2020). High-throughput screening identified mitoxantrone to induce death of hepatocellular carcinoma cells with autophagy involvement. *Biochemical and Biophysical Research Communications*.

[7] Ikeda M., Okusaka T., Sato Y. (2017). A Phase I/II trial of continuous hepatic intra-arterial infusion of 5-fluorouracil, mitoxantrone and cisplatin for advanced hepatocellular carcinoma. *Japanese Journal of Clinical Oncology*.

[8] Kroemer G., Galluzzi L., Kepp O., Zitvogel L. (2013). Immunogenic cell death in cancer therapy. *Annual Review of Immunology*.

[9] Zhou J., Wang G., Chen Y., Wang H., Hua Y., Cai Z. (2019). Immunogenic cell death in cancer therapy: present and emerging inducers. *Journal of Cellular and Molecular Medicine*.

[10] Li X. (2018). The inducers of immunogenic cell death for tumor immunotherapy. *Tumori Journal*.

[11] Krysko D. V., Garg A. D., Kaczmarek A., Krysko O., Agostinis P., Vandenabeele P. (2012). Immunogenic cell death and DAMPs in cancer therapy. *Nature Reviews Cancer*.

[12] Wang Y.-J., Fletcher R., Yu J., Zhang L. (2018). Immunogenic effects of chemotherapy-induced tumor cell death. *Genes & Diseases*.

[13] Chen Y., Zhang H., Zhang Y. (2019). Targeting receptor tyrosine kinase EphB4 in cancer therapy. *Seminars in Cancer Biology*.

[14] Kadife E., Ware T. M. B., Luwor R. B., Chan S. T. F., Nurgali K., Senior P. V. (2018). Effects of EphB4 receptor expression on colorectal cancer cells, tumor growth, vascularization and composition. *Acta Oncologica*.

[15] Rupaimoole R., Slack F. J. (2017). MicroRNA therapeutics: towards a new era for the management of cancer and other diseases. *Nature Reviews Drug Discovery*.

[16] Lee Y. S., Dutta A. (2009). Micrornas in cancer. *Annual Review of Pathology: Mechanisms of Disease*.

[17] Di Leva G., Garofalo M., Croce C. M. (2014). Micrornas in cancer. *Annual Review of Pathology: Mechanisms of Disease*.

[18] Befeler A. S., di Bisceglie A. M. (2002). Hepatocellular carcinoma: diagnosis and treatment. *Gastroenterology*.

[19] Cheng X.-l., Zhou T.-y., Li B. (2013). Methotrexate and 5-aminoimidazole-4-carboxamide riboside exert synergistic anticancer action against human breast cancer and hepatocellular carcinoma. *Acta Pharmacologica Sinica*.

[20] Yiang G.-T., Chou P.-L., Hung Y.-T. (2014). Vitamin C enhances anticancer activity in methotrexate-treated Hep3B hepatocellular carcinoma cells. *Oncology Reports*.

[21] Lv J., Xia Q., Wang J., Shen Q., Zhang J., Zhou X. (2016). EphB4 promotes the proliferation, invasion, and angiogenesis of human colorectal cancer. *Experimental and Molecular Pathology*.

[22] Bhatia S., Oweida A., Lennon S. (2019). Inhibition of EphB4-ephrin-B2 signaling reprograms the tumor immune microenvironment in head and neck cancers. *Cancer Research*.

[23] Mertens-Walker I., Lisle J. E., Nyberg W. A. (2015). EphB4 localises to the nucleus of prostate cancer cells. *Experimental Cell Research*.

[24] Garg A. D., More S, Rufo N (2017). Trial watch: immunogenic cell death induction by anticancer chemotherapeutics. *OncoImmunology*.

[25] Serrano-del Valle A., Anel A., Naval J., Marzo I. (2019). Immunogenic cell death and immunotherapy of multiple myeloma. *Frontiers in Cell and Developmental Biology*.

[26] Wang Q., Ju X., Wang J., Fan Y., Ren M., Zhang H. (2018). Immunogenic cell death in anticancer chemotherapy and its impact on clinical studies. *Cancer Letters*.

